# Quality of life after sialendoscopy: prospective non-randomized study

**DOI:** 10.1186/s12893-021-01462-2

**Published:** 2022-01-08

**Authors:** Giulianno Molina Melo, Murilo Catafesta Neves, Marcello Rosano, Christiana Maria Ribeiro Salles Vanni, Marcio Abrahao, Onivaldo Cervantes

**Affiliations:** 1 Department of Otorhinolaryngology and Surgery of Head and Neck, Federal University of São Paulo/Paulista School of Medicine (UNIFESP/EPM), R. Maestro Cardim, 560 cj 24 Bela Vista, São Paulo, SP CEP 01323000 Brazil; 2 Department of Head and Neck Surgery, Beneficencia Portuguesa of Sao Paulo Hospital, Sao Paulo, SP Brazil; 3 Department of Head and Neck Surgery, Cancer Institute of Sao Paulo-ICESP, Sao Paulo, Brazil

**Keywords:** Sialendoscopy, Sialoadenitis, Salivary gland stones, Salivary gland diseases, Quality of life

## Abstract

**Background:**

The symptomatic (swelling and pain) salivary gland obstructions are caused by sialolithiasis and salivary duct stenosis, negatively affecting quality of life (QOL), with almost all candidates for clinical measures and minimally invasive sialendoscopy. The impact of sialendoscopy treatment on the QOL has been little addressed nowadays. The objective is to prospectively evaluate the impact of sialendoscopy on the quality of life of patients undergoing sialendoscopy due to benign salivary obstructive diseases, measured through QOL questionnaires of xerostomia degree, the oral health impact profile and post sialendoscopy satisfaction questionnaires.

**Result:**

37 sialendoscopies were included, most young female; there were 64.5% sialolithiasis and 35.4% post-radioiodine; with 4.5 times/week painful swelling symptoms and 23.5 months symptom duration. The pre- and post-sialendoscopy VAS values were: 7.42 to 1.29 (p < 0.001); 86.5% and 89.2% were subjected to sialendoscopy alone and endoscopic dilatation respectively; 80.6% reported improved symptoms after sialendoscopy in the sialolithiasis clinic (p < 0.001). The physical pain and psychological discomfort domain scores were mostly impacted where sialendoscopy provided relief and improvement (p < 0.001). We found a positive correlation between sialendoscopy and obstructive stone disease (p < 0.001) and no correlation in sialendoscopy satisfaction in xerostomia patients (p = 0.009).

**Conclusions:**

We found improved symptoms with overall good satisfaction after sialendoscopy correlated with stones; and a negative correlation between xerostomia. Our findings support the evident indication of sialendoscopy for obstructive sialolithiasis with a positive impact on QOL and probably a relative time-dependent indication for stenosis/other xerostomia causes that little improved QOL satisfaction.

**Level of evidence:**

2b—Prospective non-randomized study.

*Trial registration:* WHO Universal Trial Number (UTN): U1111-1247-7028; Brazilian Clinical Trials Registry (ReBeC): RBR-6p8zfs.

## Introduction

The symptomatic obstructions of major salivary duct are primarily caused by sialolithiasis (50–75%) and salivary duct stenosis (25%) [1]; however, the annual incidence in the world can vary widely among the countries, oscillating from 1/10,000–30,000 hab. to 27–59/1,000,000 hab. [2, 3].

The etiology of obstructive sialadenitis can vary, including salivary stones, stenosis, protein plugs, anatomic variations or deformations, actinic, and autoimmune, all of which lead to salivary flow obstruction with increasing intraductal salivary pressure, swelling of the gland, and pain. It can be symptomatic, occurring mainly during meals, with a mean duration of 24–48 h, and the pain negatively affecting quality of life (QOL). Sometimes the clinical state can worsen with infection, purulent discharge, and phlogistic signals, requiring antibiotics, corticoids, anti-inflammatory drugs, fasting, and other clinical measures [1, 2, 4–8].

Anatomic variations of Wharton’s duct of the submandibular gland and saliva composition alterations play important roles in increasing the stasis of salivary flow with mucous plug and stone formation with obstructive sialolithiasis (80–90%). The parotid gland is mainly affected by salivary duct stenosis, occurring in nearly 69% of cases. It is associated with Sjögren’s disease and the radioiodine treatment of thyroid cancer [5, 9, 10]. In addition, stenosis is present in approximately 25% of all benign obstructive sialodenitis cases, caused by periductal tissue fibrosis, duct angulations, and duct gauge decrease, occurring in one or more points along the duct tree [5, 11].

The majority of obstructive salivary patients are candidates for clinical measures and endoscopic gland procedure management, because once the obstruction is removed, the gland’s function is maintained. This has been successfully achieved by minimally invasive techniques like sialendoscopy, introduced in the 1990’s for the diagnosis and treatment of salivary duct diseases. Its efficacy and utility have been proven among several authors along the years [2, 12–19]. The technique introduces a miniaturized sialendoscope (1.3 to 1.7 mm) into the opening papilla of the salivary duct, either submandibular or parotid, to inspect, clean, dilate, remove stones and infuse successfully intraglandular duct corticoids, which are utilized in nearly all benign obstructive causes [12, 14, 17, 20–28].

The quality of life during the periods of obstructive sialadenitis has been poor evaluated, with some authors demonstrating a dramatic worsening due intense face and cervical pain, feeding difficulties, weight loss, tooth and salivary problems and decreased self-care [29, 30]. Using questionnaires, the quality of life (QOL), xerostomia degree (XER) and the oral health impact profile (OHIP) has long been used to evaluate the quality of treatment in majority of head and neck cancer patients and the present authors used these previously cited to specifically evaluate the sialendoscopy treatment at the moment, although recently other papers have used different ones and some need to be worldwide validated [29–35].

The objective is to prospectively evaluate the impact of sialendoscopy on the quality of life of patients undergoing sialendoscopy due to benign obstructive diseases of the salivary glands, measured through QoL questionnaires.

It will enable us to measure the patient overall satisfaction before and after procedure; and thus, the impact of the sialendoscopy treatment, enhancing its usefulness to most centers worldwide. We hope that our results can improve the ability of assistant physician and the health system managers in better-selecting patients for sialendoscopy.

## Methods

This was a prospective, non-randomized, case series, cohort observational study, without biospecimen retention, unicentric with consecutive benign salivary gland obstructive disease patients. They were admitted and treated with sialendoscopy alone, or in combination, with a minimal cervical approach at the Department of Head and Neck Surgery between January 2017 and January 2020, with a minimum follow-up of 6 months. The inclusion criteria were as follows: consecutive patients who had undergone sialendoscopy by the same surgical team, with or without combined open facial/cervical preservative gland access as initial treatment for benign obstructive salivary gland disease; patients who agreed to participate in the study; patients who filled the formularies; and patients with indications for endoscopic treatment of salivary gland disease. The exclusion criteria included patients who were exclusively indicated for open surgery, had no indication for sialendoscopy, failed to undergo sialendoscopy during the surgical procedure, missed follow-ups, refused to complete the questionnaire or participate, had missing records, abandoned treatment prior to completion, and had previous surgery on that salivary gland or previous neck radiotherapy, due to another head and neck neoplasia.

The present study was only based on clinical data and the resulting questionnaires, without any further surgical intervention. All patients who agreed to participate in the study have written and signed the ethics approval and informed consent statement. This study was approved by the Institutional Ethics Committee (CAAE: 95881418.2.0000.5483, number 2.934.247) in October 2018.

The study was conducted in accordance with the Declaration of Helsinki and registered with the WHO Universal Trial Number (UTN) number (U1111-1247-7028) and the Brazilian Clinical Trials Registry (ReBeC), whose number is RBR-6p8zfs. This study is in accordance with the Preferred Reporting of Case Series in Surgery (PROCESS) criteria [36], Strengthening the Reporting of Cohort Studies in Surgery (STROCSS) [37] and the Standards for Quality Improvement Reporting Excellence guidelines (SQUIRE 2.0) [38].

### Study design

All eligible consecutive patients with benign salivary gland obstructive disease patients who will be treated with sialendoscopy alone (or in possible combination with a minimal cervical approach as necessary), after provided their consent to participate in the study, were given the QOL questionnaires and the visual pain analog scale (VAS) just before the procedure; and again after 2 months prospectively after sialendoscopy procedure, where they were called by phone, filled out the forms and brought them to follow-up consultations.

### Questionnaires

The QOL questionnaires applied were as follows:

The OHIP questionnaire—the oral health impact profile, as validated to Brazilian portuguese language, [39–41] with 49 questions on seven domains: functional limitation, physical pain, psychological discomfort, physical disability, psychological disability, social disability and handicap; that measures people's perceptions of the impact of oral conditions on their well-being. Responses from the patients were made on a Likert-type scale, coded as: 0 = never; 1 = hardly ever; 2 = occasionally; 3 = fairly often and 4 = very often; the descriptive statistics were created by computing the mean of each coded response.

The Xerostomia (Xer) questionnaire, also validated in Brazilian Portuguese language [31, 42, 43] with 21 questions covering the symptomatic obstructive, hyposalivation and inflammatory salivary pathologies related to salivary gland dysfunction. The patient has to score from 1 to 5 each condition, according to the severity, so a higher score correspond to an increased complaint.

The questionnaire on patient satisfaction post-sialendoscopy (PSPS) [30] was created based on the existing quality of life surveys, with 14 questions covering the main afflictions before and after the sialendoscopy procedure and the overall satisfaction. This survey included dichotomous response choices and ten-level response choice, scaled from 1 to 10.

The visual pain analog scale (VAS) [44] for pain analysis, consisting of a straight line with the endpoints defining the limits, with “no pain at all” and “pain bad as could be”; the scale is numerically rated from 0 to 10 and the patients are asked to circle the number from 1 to 10 or the face figure drawing corresponding to the symptoms.

Clinical and demographic data, sialendoscopy diagnostic and intervention data results, and data from the questionnaires (OHIP, Xer, and PSPS) were collected. Follow-up was performed with regular consultations, one week after post-operatory procedures and then at 30, 60, and 90 days, with a salivary gland ultrasonography (USG) at 90 days in all patients.

All data were collected and the statistical analyses were performed using the Spearman’s correlation test, Mann–Whitney test, two-proportions equality test, Wilcoxon test, and chi-square test, with a significance of p < 0.05.

### Sialendoscopy protocol

**Fig. 1:**
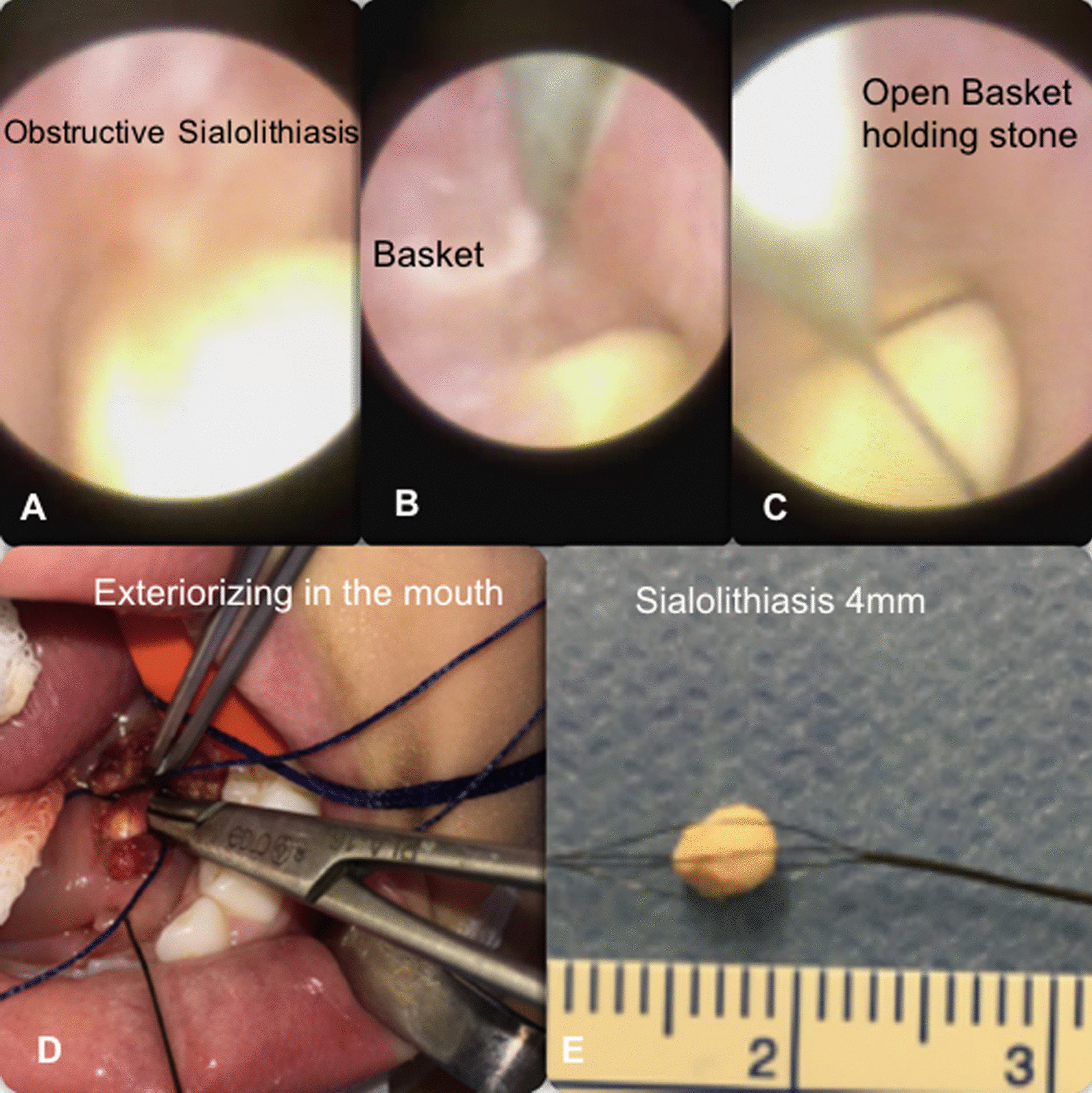
Final image stone sequence. **A** Obstructive sialolithiasis in the main duct. **B** Basket in position beside the stone. **C** Open basket holding the stone. **D** Exteriorizing the set through the mouth. **E** Sialolithiasis measuring 4 mm

**Fig. 2:**
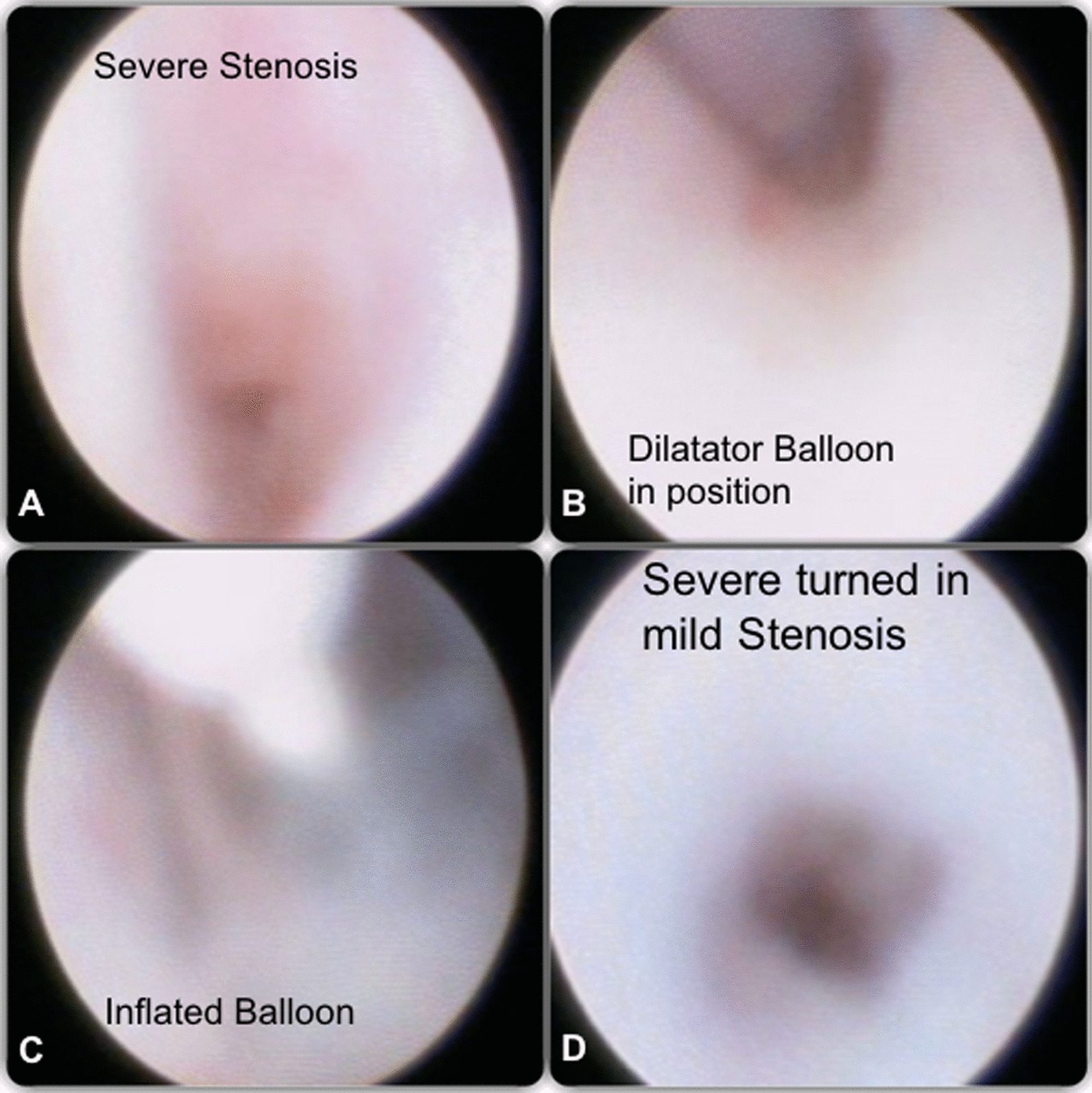
Final image stenosis sequence. **A** Severe Stenosis with pale intraductal mucosa. **B** Dilatator Balloon in position, inside the stenosis. **C** Inflated Balloon, one can see the light reflect in the balloon filled with water. **D** Severe turned in mild Stenosis improving the saliva flow

Sialendoscopy was performed by the same surgeon (GMM) and surgical team, following the Marchal et al*.* standards [14] in the operating room, in-hospital, and under general anesthesia. It was performed on the involved gland for both diagnostic and therapeutic interventions, using the semi-rigid modular sialendoscope (Karl Storz, Tuttlingen, Germany) (diameter 1.3 mm or 1.7 mm), with working channel, salivary probes, conic dilatators, bougies, baskets for stones, dilatator balloons, silastic stents to the main duct, and papilla patency [27] (Figs. 1, 2). No case with acute purulent salivary discharge and sialadenitis were submitted to the procedure.

In cases of minimal open access, a 2–3 cm skin incision was made in the appropriate skin crease. Minimal surgical dissection techniques were achieved with facial nerve monitoring; the main duct was opened and the impacted stone was removed. All cases were subjected to intraductal steroids delivered with the sialendoscope, main duct and papilla stenting with silicone, and were withdrawn after 21 days. All patients remained in-hospital for at least 24 h and were discharged to ambulatory follow-up.

## Results

During this period of three years, 40 patients underwent sialendoscopy. Five refused to participate and four missed follow-up appointments. The final cohort included 37 sialendoscopies in 31 patients. All patients underwent preoperative examinations with at least salivary gland ultrasound, computed tomography (CT), and magnetic resonance imaging.

### Clinical data

The population was comprised of 17 females and 14 males with a mean age of 44.7 years (11–80 years) and follow-up of 14 months (6–38 months). Clinical characteristics are shown in Table 1. Frequent comorbidities included hypertension (29.0%), previous radioiodine treatment (16.1%), and 19.35% high-volume milk ingestion (> 1.000 mL/day). No case with acute purulent salivary discharge and sialadenitis were submitted to the procedure.

**Table 1:** Clinical and symptoms characteristics

Clinical characteristics	N	Percent
	31	100%
Gender		
Male	14	45.20%
Female	17	54.80%
Age (years) (average/ range)	44.7	Nov-80
Follow-up (months) (average/ range)	14	Jun-26
Comorbidities		
Hypertension	9	29.00%
Diabetes mellitus	2	3.20%
Auto-immune diseases	2	6.45%
Thyroid cancer with RIT	5	16.10%
Tobacco smoker	2	6.45%
High volume milk ingestion	6	19.35%
Antidepressant medication	2	6.45%
Time to diagnosis at first consultation (months)	23.2	1–168
(average/range)		
Symptoms characteristics before procedure (more than one)		
Swellings	30	96,8%
Pain	28	90.30%
Pus in the oral cavity	6	19.35%
Sialolithiasis perception	18	58.00%
Salivation changes	14	45.20%
Dry mouth	10	32.20%
Time of symptoms duration (months) (average, range)	23.5	1–168
Complaints per week (average, range)	4.5	Jan-14
Pre-operatory pain (VAS 0–10)	7.4	01-Oct
(average/ range)		
Gland involved		
Parotid	10	32.30%
Submandibular	21	67.70%
Sublingual	0	0%
Side		
Right	17	54.80%
Left	8	25.80%
Bilateral	6	19.40%
Etiology (some bilateral)		
Pure stones	16	51.60%
Stenosis (radioiodine/inflammatory)	13	41.90%
Stenosis + stones	4	12.90%
Radiological pre-operatory exam		
Ultrasound (USG)	30	96.70%
Tomography (CT)	18	58.10%
Resonance (MR)	14	45.20%
Scintigraphy	5	16.10%
Size stones on USG (mm) (average/ range)	3.77	Feb-15

*VAS* pain Visual Analogic Scale

Swelling (96.8%) and pain (90.3%) were the most frequent symptoms, with an average complaint rate of 4.5 times per week, pre-pain VAS average of 7.42 (1–10), delay of 21.7 months from first symptom to medical diagnosis, and symptom duration of 23.5 months (1–168 months). No patients required resection surgery (Table 1). The involved glands were the submandibular (67.7%) and parotid (32.3%), right side (54.8%) and bilateral in 19.4% of cases. The etiology was as follows: stones, 51.6%; stenosis, 41.9%; preoperative USG, 96.7%; CT, 58.1%. The average intraductal stone size on USG was 3.77 mm (2–15 mm).

### Clinical characteristics of sialendoscopy

Table 2 shows the sialendoscopy findings, 86.5% were subjected to sialendoscopy alone, 89.2% to endoscopic dilatation, and 100% to intraductal steroids. The percentages according to diagnoses were as follows: 48.6% submandibular stone, 40.5% pure stones, and 32.4% papilla stenosis. The most common papilla type was type A (48.6%). Stenting (papilla or duct, once it is difficult to only stent the papilla site): 100% and dilatation (35.1%) were the most common procedures. The stones were single in 37.8% of cases, overall complications were 10.8%, average time of sialendoscopy was 139.5 min, and the postoperative pain score was 1.3. All patients submitted to the combined-hybrid procedure have answered the questionnaires with the main objective of evaluating the role of sialendoscopy associated or not with the combined procedure.

**Table 2:** Sialendoscopy clinical characteristics

Sialendoscopy clinical characteristics	N	Percent
	37	100%
Sialendoscopy alone	32	86.50%
Combined sialendoscopy	5	13.50%
Sialendoscopy procedures (more than one)		
Endoscopic stone extraction	20	54.10%
Endoscopic dilatation	33	89.20%
Intraductal stenting	30	81.10%
Intraductal steroids	37	100%
Sialendoscopy diagnosis verified (more than one)		
Pure stones	15	40.50%
Parotid stones	3	8.10%
Submandibular stones	18	48.60%
Stones and stenosis	4	10.80%
Papilla stenosis	12	32.40%
Parotid duct stenosis	9	24.30%
Submandibular duct stenosis	5	13.50%
Papilla types		
A	18	48.60%
B	7	18.90%
C	2	5.40%
D	4	10.80%
E	6	16.20%
Procedures on papilla (more than one)		
Papillotomy	10	27.00%
Dilatation	13	35.10%
Opening floor of mouth	10	27.00%
Marsupialization	12	32.40%
Stenting	37	100%
Stones characteristics		
Single	14	37.80%
Multiple	6	16.20%
Post-operative complications		
Lost stent	4	10.80%
Infection	0	0%
Dehiscence	0	0%
Endoscopic duct classification LSD		
L0	16	43.20%
L1	12	32.40%
L2	4	10.80%
L3	5	13.50%
S0	17	45.90%
S1	13	35.10%
S2	3	8.10%
S3	3	8.10%
S4	1	2.70%
D0	17	45.90%
D1	11	29.70%
D2	9	24.30%
D3	0	0%
Complications	4	10.80%
Sialendoscopy time duration (min)		
(average, range)	139.5	80–210
Post-operatory Pain (VAS 0–10)		
(average/ range)	1.3	0–3

Bold values indicate the findings of statistical significance

*LSD* lithiasis, stenosis and dilatation endoscopic classification, *VAS* pain Visual Analogic Scale

### Questionnaire findings

#### Patient satisfaction post-sialendoscopy—(PSPS questionnaire)

In our findings, the most important question on the PSPS questionnaire, given after the procedure, was number 7 (Sialo7), which indicated the overall satisfaction of the patient with sialendoscopy, the other questions were equal to prior questionnaires; we coded the answers with numbers 1 to 4: Bad (1), Satisfactory (2), Good (3), and Very Good (4). The average was 3.45, indicating that the majority of patients expressed Very Good/Good satisfaction with sialendoscopy. We compared all the other questionnaires with the answer Sialo7.

#### Oral health impact profile (OHIP) and Xerostomia (Xer) questionnaires

The overall OHIP punctuation was 32.52 ± 10.82 (196 total points). The Xer questionnaire, the overall average was 24.65 ± 7.06 (105 total points).

Table 3 demonstrates the frequency of qualitative clinical data, showing statistical differences in diabetes mellitus, hypertension, autoimmune diseases, high milk ingestion, tobacco, submandibular and parotid gland, on-bulking, dry mouth, salivary lithiasis, salivary changes, and right side. For the Sialo7 question, 80.6% were Very Good/Good versus 19.4% Satisfactory/Bad (p < 0.001).

**Table 3:** Frequency distribution of qualitative clinical data

		N	%	P-valor
Comorbidities	No	16	51.6	0.799
	Yes	15	48.4	
DM	No	28	93.3	** < 0.001**
	Yes	2	6.7	
Autoimmune disease	No	28	93.3	** < 0.001**
	Yes	2	6.7	
Actual salivary gland: parotid	No	21	67.7	**0.005**
	Yes	10	32.3	
Actual salivary gland: submand	No	10	32.3	**0.005**
	Yes	21	67.7	
Hypertension	No	21	70.0	**0.002**
	Yes	9	30.0	
Milk ingestion	No	23	79.3	** < 0.001**
	Yes	6	20.7	
Other	No	22	78.6	** < 0.001**
	Yes	6	21.4	
Gender	Female	18	58.1	0.204
	Male	13	41.9	
Sialo 7	Very/Good	25	80.6	** < 0.001**
	Satisf./Bad	6	19.4	
Tobacco use	No	28	96.6	** < 0.001**
	Yes	1	3.4	
Symptom: swelling	No	1	3.2	** < 0.001**
	Yes	30	96.8	
Symptom: dry mouth	No	21	67.7	**0.005**
	Yes	10	32.3	
Symptom: salivary stone	No	11	35.5	**0.022**
	Yes	20	64.5	
Symptom: saliva changes	Não	15	48,4%	0.799
	Sim	16	51,6%	
Compromised side	Bilateral	6	19.4	**0.004**
	Rigth	17	54.8	**Ref**
	Left	8	25.8	**0.020**

Bold values indicate the findings of statistical significance

*DM* diabetes mellitus

Sialo 7: question 7 in the Patient satisfaction post-sialendoscopy questionnaire—(PSPS questionnaire) meaning the overall satisfaction of the patient with sialendoscopy

Table 4 shows Spearman’s correlation for the Sialo7 question (major satisfaction), relating satisfaction with the sialendoscopy procedure to the variables mentioned. When positive, the correlated variables increased proportionally; however, when the correlation was negative, it implied that the variables were inversely proportional.

**Table 4:** Correlation of PSPS (question Sialo 7) with ordinal and quantitative variables

		Sialo (Q7)	
		Corr (r)	P-valor
Demographics	Age	− 0.293	0.110
	Time to diagnosis	− 0.210	0.257
	Symptoms time	− 0.165	0.376
	Pre VAS	− 0.194	0.296
	Symptoms frequency	0.170	0.360
	USG stone size	0.357	**0.049**
Functional limitation	Q1	− 0.040	0.830
	Q2	− 0.140	0.451
	Q3	− 0.376	**0.037**
	Q4	− 0.032	0.865
	Q5	− 0.303	0.097
	Q6	− 0.218	0.238
	Q7	0.048	0.797
	Q8	− 0.042	0.823
	Q17	− 0.296	0.106
	Functional limitation	− 0.080	0.669
Physical pain	Q9	− 0.167	0.369
	Q10	0.124	0.505
	Q11	0.113	0.544
	Q12	− 0.134	0.474
	Q13	− 0.201	0.278
	Q14	− 0.015	0.936
	Q15	0.094	0.613
	Q16	− 0.152	0.413
	Q18	− 0.349	0.055
	Physical pain	0.107	0.567
Psychological discomfort	Q19	− 0.188	0.311
	Q20	− 0.188	0.311
	Q21	− 0.605	** < 0.001**
	Q22	0.009	0.964
	Q23	− 0.093	0.618
	Psychological discomfort	− 0.235	0.204
Physical disability	Q24	− 0.269	0.143
	Q25	− 0.398	**0.026**
	Q26	− 0.119	0.523
	Q27	− 0.099	0.598
	Q28	0.104	0.577
	Q29	0.043	0.818
	Q30	− 0.349	0.055
	Q31	− 0.271	0.140
	Q32	0.006	0.973
	Physical disability	− 0.081	0.666
Psychological disability	Q33	− 0.159	0.392
	Q34	− 0.118	0.528
	Q35	− 0.203	0.274
	Q36	− 0.389	**0.031**
	Q37	− 0.102	0.586
	Q38	0.010	0.959
	Psychological disability	− 0.089	0.634
Social disability	Q39	− 0.162	0.385
	Q40	− 0.073	0.698
	Q41	− 0.324	0.075
	Q42	− 0.206	0.266
	Q43	− 0.134	0.472
	Social disability	− 0.123	0.508
Handicap	Q44	− 0.287	0.118
	Q45	− 0.478	**0.006**
	Q46	− 0.660	** < 0.001**
	Q47	− 0.441	**0.013**
	Q48	− 0.349	0.055
	Q49	− 0.296	0.106
	Handicap	− 0.465	**0.008**
Total OHIP		− 0.111	0.554
Xerostomia	P1	0.004	0.985
	P2	0.046	0.805
	P3	− 0.188	0.312
	P4	− 0.306	0.094
	P5	− 0.403	**0.025**
	P6	− 0.388	**0.031**
	P7	− 0.301	0.100
	P8	− 0.254	0.169
	P9	− 0.310	0.090
	P10	− 0.364	**0.044**
	P11	− 0.283	0.123
	P12	− 0.244	0.186
	P13	− 0.274	0.135
	P14	− 0.390	**0.030**
	P15	− 0.443	**0.013**
	P16	− 0.242	0.189
	P17	− 0.334	0.066
	P18	− 0.509	**0.003**
	P19	− 0.203	0.272
	P20	− 0.259	0.159
	P21	− 0.301	0.100
	Total	− 0.284	0.122

Bold values indicate the findings of statistical significance

Sialo 7: question 7 in the Patient satisfaction post-sialendoscopy questionnaire—(PSPS questionnaire) meaning the overall satisfaction of the patient with sialendoscopy

*OHIP* oral health impact profile questionnaire, *Xerostomia* Xerostomia (Xer) questionnaires

We found a positive correlation between sialendoscopy and calculi size: the amount of sialolithiasis associated with better sialendoscopy satisfaction results. The best correlation was with question 46 of OHIP, which showed that the higher the Sialo7 (the greater the satisfaction), the lower the question 46 score, which was classified as Very Good.

Table 5 demonstrates the grouped answers of Sialo7 in Very Good/Good and Satisfying/Bad in the Mann–Whitney test to compare the quantitative variables in the various groups. There were differences in the OHIP: question 17 (p = 0.041), question 45 (p = 0.014), question 46 (p = 0.002), and Xer total score (p = 0.009). These results showed no correlation in sialendoscopy satisfaction in xerostomia patients, where the mean of Satisfying/Bad was 46.5 versus 19.4 Very Good/Good answers (p = 0.009).

**Table 5:** Comparison of the PSPS (question 7) with ordinal and quantitative variables and to the oral health impact profile (OHIP) and Xerostomia (Xer) questionnaires

		Average	Median	Standard deviation	N	IC	P-valor
Age	Satisf./Bad	50.8	53.5	12.4	6	9.9	0.202
	Very/Good	43.3	40	16.2	25	6.3	
Time to diagnosis	Satisf./Bad	30.8	13	49.7	6	39.8	0.192
	Very/Good	19.6	8	38.0	25	14.9	
Symptoms time	Satisf./Bad	20.5	19.5	7.8	6	6.2	0.260
	Very/Good	24.3	12	36.6	25	14.3	
Pre VAS	Satisf./Bad	8.00	8	1.90	6	1.52	0.446
	Very/Good	7.28	7	2.09	25	0.82	
Symptoms frequency	Satisf./Bad	4.17	3	2.23	6	1.78	0.879
	Very/Good	4.60	4	3.21	25	1.26	
USG stone size	Satisf./Bad	1.67	0	3.20	6	2.56	0.089
	Very/Good	4.28	4	4.15	25	1.63	
Q1	Satisf./Bad	1.00	0.5	1.26	6	1.01	0.826
	Very/Good	0.92	0	1.26	25	0.49	
Q2	Satisf./Bad	0.17	0	0.41	6	0.33	0.534
	Very/Good	0.36	0	0.64	25	0.25	
Q3	Satisf./Bad	0.50	0	0.84	6	0.67	0.274
	Very/Good	0.36	0	1.11	25	0.44	
Q4	Satisf./Bad	0.83	0,5	0.98	6	0,79	0.596
	Very/Good	0.68	0	1.07	25	0,42	
Q5	Satisf./Mal	0.33	0	0.82	6	0.65	0.731
	Very/Good	0.20	0	0.58	25	0.23	
Q6	Satisf./Bad	1.00	0	1.67	6	1.34	0.277
	Very/Good	0.32	0	0.80	25	0.31	
Q7	Satisf./Bad	0.50	0	1.22	6	0.98	0.785
	Very/Good	0.56	0	1.12	25	0.44	
Q8	Satisf./Bad	0.83	0	1.33	6	1.06	0.595
	Very/Good	0.44	0	0.82	25	0.32	
Q17	Satisf./Bad	0.33	0	0.82	6	0.65	**0.041**
	Very/Good	0.00	0	0.00	25	- x -	
Functional limitation	Satisf./Bad	5.50	2	7.84	6	6.27	0.980
	Very/Good	3.84	2	3.91	25	1.53	
Q9	Satisf./Bad	1.33	0	2.07	6	1.65	0.630
	Very/Good	0.68	0	1.18	25	0.46	
Q10	Satisf./Bad	1.17	0.5	1.60	6	1.28	0.562
	Very/Good	1.56	2	1.45	25	0.57	
Q11	Satisf./Bad	0.67	0	1.63	6	1.31	0.271
	Very/Good	0.88	1	1.01	25	0.40	
Q12	Satisf./Bad	0.67	0	1.63	6	1.31	0.971
	Very/Good	0.52	0	1.16	25	0.45	
Q13	Satisf./Bad	0.50	0	1.22	6	0.98	0.876
	Very/Good	0.24	0	0.66	25	0.26	
Q14	Satisf./Bad	0.50	0	1.22	6	0.98	0.696
	Very/Good	0.48	0	0.92	25	0.36	
Q15	Satisf./Bad	1.33	0.5	1.75	6	1.40	0.671
	Very/Good	1.76	2	1.71	25	0.67	
Q16	Satisf./Bad	0.50	0	1.22	6	0.98	0.672
	Very/Good	0.64	0	1.25	25	0.49	
Q18	Satisf./Bad	0.50	0	1.22	6	0.98	0.240
	Very/Good	0.04	0	0.20	25	0.08	
Physical pain	Satisf./Bad	7.17	1	12.66	6	10.13	0.248
	Very/Good	6.80	6	5.93	25	2.32	
Q19	Satisf./Bad	2.17	2	1.17	6	0.94	0.959
	Very/Good	2.04	2	1.40	25	0.55	
Q20	Satisf./Bad	1.50	1.5	1.38	6	1.10	0.834
	Very/Good	1.48	1	1.71	25	0.67	
Q21	Satisf./Bad	1.00	0.5	1.26	6	1.01	**0.060**
	Very/Good	0.24	0	0.66	25	0.26	
Q22	Satisf./Bad	1.33	1.5	1.21	6	0.97	0.916
	Very/Good	1.36	1	1.50	25	0.59	
Q23	Satisf./Bad	1.50	1.5	1.38	6	1.10	0.959
	Very/Good	1.52	2	1.42	25	0.56	
Psychological discomfort	Satisf./Bad	7.50	7	4.93	6	3.94	0.598
	Very/Good	6.64	7	4.51	25	1.77	
Q24	Satisf./Bad	0.17	0	0.41	6	0.33	0.864
	Very/Good	0.32	0	0.90	25	0.35	
Q25	Satisf./Bad	0.17	0	0.41	6	0.33	0.526
	Very/Good	0.08	0	0.28	25	0.11	
Q26	Satisf./Bad	0.83	0	1.60	6	1.28	0.493
	Very/Good	0.40	0	0.82	25	0.32	
Q27	Satisf./Bad	0.17	0	0.41	6	0.33	0.830
	Very/Good	0.28	0	0.89	25	0.35	
Q28	Satisf./Bad	0.67	0	1.63	6	1.31	0.696
	Very/Good	0.76	0	1.36	25	0.53	
Q29	Satisf./Bad	1.00	0	1.67	6	1.34	0.906
	Very/Good	0.76	0	1.16	25	0.46	
Q30	Satisf./Bad	0.50	0	1.22	6	0.98	0.240
	Very/Good	0.04	0	0.20	25	0.08	
Q31	Satisf./Bad	0.50	0	1.22	6	0.98	0.830
	Very/Good	0.44	0	1.23	25	0.48	
Q32	Satisf./Bad	1.00	0.5	1.26	6	1.01	0.956
	Very/Good	1.04	0	1.34	25	0.52	
Physical disability	Satisf./Bad	5.00	1	9.42	6	7.54	0.917
	Very/Good	4.12	2	5.42	25	2.12	
Q33	Satisf./Bad	0.83	0	1.33	6	1.06	1.000
	Very/Good	0.80	0	1.35	25	0.53	
Q34	Satisf./Bad	1.33	1	1.37	6	1.09	0.874
	Very/Good	1.32	2	1.31	25	0.52	
Q35	Satisf./Bad	1.17	0.5	1.47	6	1.18	0.872
	Very/Good	1.04	1	1.17	25	0.46	
Q36	Satisf./Bad	1.17	0.5	1.60	6	1.28	0.290
	Very/Good	0.48	0	0.82	25	0.32	
Q37	Satisf./Bad	1.17	0	1.83	6	1.47	1.000
	Very/Good	0.96	0	1.27	25	0.50	
Q38	Satisf./Bad	0.67	0	1.63	6	1.31	0.640
	Very/Good	0.60	0	1.00	25	0.39	
Psychological disability	Satisf./Bad	6.33	4	8.14	6	6.51	0.801
	Very/Good	5.20	5	4.25	25	1.67	
Q39	Satisf./Bad	0.67	0	1.63	6	1.31	1.000
	Very/Good	0.44	0	0.96	25	0.38	
Q40	Satisf./Bad	0.83	0	1.60	6	1.28	0.930
	Very/Good	0.76	0	1.16	25	0.46	
Q41	Satisf./Bad	0.83	0	1.60	6	1.28	0.328
	Very/Good	0.32	0	0.80	25	0.31	
Q42	Satisf./Bad	1.17	0.5	1.60	6	1.28	0.447
	Very/Good	0.76	0	1.27	25	0.50	
Q43	Satisf./Bad	0.50	0	1.22	6	0.98	0.240
	Very/Good	0.04	0	0.20	25	0.08	
Social disability	Satisf./Bad	4.00	1	7.46	6	5.97	0.742
	Very/Good	2.32	0	3.69	25	1.45	
Q44	Satisf./Bad	1.33	1	1.63	6	1.31	0.251
	Very/Good	0.52	0	0.77	25	0.30	
Q45	Satisf./Bad	1.33	1	1.63	6	1.31	**0.014**
	Very/Good	0.20	0	0.71	25	0.28	
Q46	Satisf./Bad	1.50	1.5	1.52	6	1.21	**0.002**
	Very/Good	0.12	0	0.33	25	0.13	
Q47	Satisf./Bad	1.17	0.5	1.60	6	1.28	0.189
	Very/Good	0.44	0	0.87	25	0.34	
Q48	Satisf./Bad	0.50	0	1.22	6	0.98	0.240
	Very/Good	0.08	0	0.40	25	0.16	
Q49	Satisf./Bad	0.50	0	1.22	6	0.98	**0.041**
	Very/Good	0.00	0	0.00	25	- x -	
Handicap	Satisf./Bad	6.33	4	8.33	6	6.67	0.085
	Very/Good	1.36	0	2.31	25	0.90	
Total OHIP	Satisf./Bad	57.17	50.5	52.94	6	42.36	0.193
	Very/Good	26.60	23	20.24	25	7.93	
P1	Satisf./Bad	2,67	2,5	1,86	6	1,49	0,174
	Very/Good	1,64	1	1,58	25	0,62	
P2	Satisf./Bad	2.67	2.5	1.86	6	1.49	0.257
	Very/Good	1.84	1	1.65	25	0.65	
P3	Satisf./Bad	2.67	3	1.37	6	1.09	**0.013**
	Very/Good	1.04	1	1.24	25	0.49	
P4	Satisf./Bad	2.50	1.5	1.97	6	1.58	**0.033**
	Very/Good	0.92	1	0.91	25	0.36	
P5	Satisf./Bad	3.17	3.5	2.04	6	1.63	**0.009**
	Very/Good	1.04	1	1.10	25	0.43	
P6	Satisf./Bad	2.33	1	2,07	6	1.65	**0.009**
	Very/Good	0.60	1	0.50	25	0.20	
P7	Satisf./Bad	2.50	2	1.76	6	1.41	**0.015**
	Very/Good	0.84	1	1.11	25	0.43	
P8	Satisf./Bad	2.33	2.5	1.21	6	0.97	**0.014**
	Very/Good	0.96	1	1.27	25	0.50	
P9	Satisf./Bad	3.00	3	2.19	6	1.75	**0.006**
	Very/Good	0.72	1	1.06	25	0.42	
P10	Satisf./Bad	1.83	1	1.33	6	1.06	**0.009**
	Very/Good	0.64	0	1.04	25	0.41	
P11	Satisf./Bad	2.17	2	1.33	6	1.06	0.075
	Very/Good	1.16	1	1.21	25	0.48	
P12	Satisf./Bad	2.33	2	1.51	6	1.20	**0.011**
	Very/Good	0.80	1	1.00	25	0.39	
P13	Satisf./Bad	1.50	1	1.22	6	0.98	0.072
	Very/Good	0.68	1	0.69	25	0.27	
P14	Satisf./Bad	2.17	1	1.83	6	1.47	0.103
	Very/Good	0.96	1	0.98	25	0.38	
P15	Satisf./Bad	1.67	1	1.63	6	1.31	**0.027**
	Very/Good	0.60	0	0.76	25	0.30	
P16	Satisf./Bad	2.00	1	1.67	6	1.34	0.143
	Very/Good	1.16	1	1.34	25	0.53	
P17	Satisf./Bad	1.50	1	1.22	6	0.98	0.088
	Very/Good	0.88	1	1.20	25	0.47	
P18	Satisf./Bad	1.50	1	0.84	6	0.67	**0.018**
	Very/Good	0.64	1	0.76	25	0.30	
P19	Satisf./Bad	2.00	1	1.67	6	1.34	**0.044**
	Very/Good	0.84	1	1.03	25	0.40	
P20	Satisf./Bad	2.17	1.5	1.60	6	1.28	**0.011**
	Very/Good	0.72	1	0.79	25	0.31	
P21	Satisf./Bad	1.83	1	1.33	6	1.06	**0.027**
	Very/Good	0.72	1	0.79	25	0.31	
Total	Satisf./Bad	46.50	42.5	24.34	6	19.47	**0.009**
	Very/Good	19.40	23	15.19	25	5.96	

Sialo 7: question 7 in the Patient satisfaction post-sialendoscopy questionnaire – (PSPS questionnaire) meaning the overall satisfaction of the patient with sialendoscopy

*OHIP* oral health impact profile questionnaire—Q1 to Q49

*Xerostomia* Xerostomia (Xer) questionnaires—P1 to P21

*VAS* pain Visual Analogic Scale

The comparison of pre- and post-sialendoscopy VAS values (Wilcoxon test) resulted in a score reduction from 7.42 to 1.29 (p < 0.001), showing the efficacy of sialendoscopy in relieving pain after treatment.

## Discussion

### Synopsis of new findings

This prospective study evaluated the post-sialendoscopy satisfaction by QOL questionnaire results for 37 sialendoscopies in three years. Few studies have focused specifically on the QOL after sialendoscopies; previous specific questionnaires, like the Chronic Obstructive Sialadenitis Symptoms (COSS) Questionnaire [45], have retrospectively addressed the severity of sialadenitis symptoms in sialendoscopy submitted patients, in seven years period with only 66 patients enrolled and, different from our study, they evaluated a past month clinical period.

Our study differs in the complete and prospective way in which the topic was addressed by specific questionnaires of sialendoscopy, xerostomia and OHIP, before and after the procedure, with a good correlation of the result with sialendoscopy, with findings similar to another prospective study with forty patients and specific questionnaire [29] and to date, there are no other comparable studies, despite the growing spread of the technique [31].

Our cohort included most young female patients: 64.5% had sialolithiasis, 35.4% had post-radioiodine; the periodic painful swelling (4.5 times/week), and a long average time until treatment (23.5 months) could have strongly influenced the poor pre-sialendoscopy QOL, once the pre-VAS was 7.42 (p < 0.001). This was anatomically explained by the sensitive gland innervation from trigeminal V3 branches. Our post-sialendoscopy follow-up (14 months) confirmed the successful viability of the sialendoscopy as an organ function-preserving procedure, with a high satisfaction index.

In our cohort, 64.5% of patients suffered from stones obstruction with an average size of 3.77 mm. Nearly 37% were single stones of which 86.5% were successfully treated with sialendoscopy alone, and the remaining with a combined approach. The average time of 139 min (2 h and 31 min), without complications, was comparable with the literature, in the way that some patients (majority with stones and five others with combined-hybrid procedure), have took more time to retrieve the objective, and they took part of the first cases of the study, being interpreted also as a biases; nowadays it took about forty five minutes [5, 46–48]. The post-VAS pain scale was 1.3 after sialendoscopy (p < 0.001). There was major satisfaction with the procedure, as 3.45 was the overall satisfaction score (p < 0.001), which mainly correlated with stone size (p = 0.049) and was comparable with only one other similar article [29] (Tables 1, 2).

#### Oral health impact profile and sialendoscopy findings

Overall, 80.6% of patients reported improved symptoms after sialendoscopy in the sialolithiasis clinic (p < 0.001) (Table 3). In the OHIP, the physical pain and psychological discomfort domain scores were mostly impacted by salivary obstruction. As these QOL domains were heavily impacted by obstruction, the sialendoscopy provided relief and truly improved psychological discomfort and physical and psychological deficiencies (p < 0.001) (Table 4), similar to recent studies [32–34].

Our study limitations were the relatively small number of patients for this amount of time; questionable conclusions due to the interpretation of subjective data on QOL questionnaires, common in this type of studies; the absence of comparative results in literature to ours of specific questionnaires on sialendoscopy; and patient misinterpretation with different types of questions. Nevertheless, our prospective study on post-sialendoscopy satisfaction found high score QOL correlated with stone size.

In our correlation analysis (Table 4), we found a positive correlation with calculi size, that is, larger sialolithiasis and better sialendoscopy satisfaction (p = 0.049). We found the best correlation with question 46 (unable to enjoy people's company) of OHIP, where r = − 0.660. This negative r-correlation shows, inversely, a greater satisfaction with sialendoscopy, as demonstrated in the QOL questionnaire.

In Table 3, the salivary stone symptom correlated with Good satisfaction (p = 0.022) and overall Good satisfaction with sialendoscopy for obstructive disease (p < 0.001), demonstrating the efficacy of sialendoscopy in relieving pain and an enriching QOL.

In Table 4, other significant Very Good correlations of sialendoscopy included the following: OHIP: questions 3, 21, 25, 36, 45, 47, and total deficiency, meaning that OHIP questions prior to sialendoscopy (such as tooth problems, psychological discomfort, depression, and an unsatisfying life) have a strong correlation with Very Good satisfaction after sialendoscopy procedure. This mainly reflects the patient mental status improvement after relief of pain and resolution of the obstructive salivary problems.

Similar results are shown in Table 5, with respect to the satisfaction answer: Very Good/Good and Satisfying/Bad. The main differences occurred on question 17 (p = 0.041), question 45 (p = 0.014), and question 46 (p = 0.002), implying good correlation after the sialendoscopy, in which the procedure ameliorated in some way the prior symptoms.

#### Xerostomia and sialendoscopy findings

We found good correlation between sialendoscopy satisfaction in Q5 (p = 0.025), Q6 (p = 0.031), Q10 (p = 0.044), Q14 (p = 0.030), Q15 (p = 0.013), and Q18 (p = 0.003) (Table 4). This showed that worries prior to the procedure were positively associated with resolution and satisfaction after sialendoscopy. These findings lead to conclude that when the patient felling of xerostomia were mainly due stenosis problems of various etiologies, the sialendoscopy are the main mean of treatment, similarly to others studies [33, 49].

However, in Table 5, we found a negative correlation between Xer and sialendoscopy satisfaction, where the total score was 46.5 Satisfying/Bad versus Very Good/Good (p = 0.009). These results demonstrated no correlation in sialendoscopy satisfaction, similar to the literature, specifically on cases of mixture of secondary or main duct stenosis, radioiodine treatment for thyroid cancer, and salivary production deficiency, common findings in auto-immune diseases, diabetes mellitus, tobacco smoker and antidepressant medication users [8, 11, 20, 25, 26, 31].

These contradictory results could be explained by the fact that the main disease that determined the stenosis is the same on salivary tissue and acini destruction. As the Poiseuille’s law, these alterations (quality of saliva as viscosity, volume of saliva production determining the pressure gradient across the tubing, duct length and duct diameter) altogether contribute to decreased salivary production and flow; and since the sialendoscopy is a procedure that ameliorate the diameter of the duct, facilitating the saliva flow, it affects only the flow part of equation [50]. Everything else of the salivary production is not achieved and solved by sialendoscopy, and therefore, the final result is the poor satisfaction expressed by patients along time [30, 33]; other explanations are patient misunderstanding, method limitations and the relatively few subjects on the study.

### Clinical applications

Our findings support the evident first indication of sialendoscopy for obstructive sialolithiasis treatment and probably a relative time-dependent indication for stenosis/other xerostomia causes due the re-incident nature of the strictures. The positive impact on QOL is clearly evident on the sialolithiasis and barely satisfactory in the stenosis; as result, the surgeon must precisely evaluate the time of each case indication.

The positive satisfaction of sialendoscopy for pain relief in obstructive disease, mainly due to stones while conserving the salivary gland, reaffirms the indication of sialendoscopy as the first alternative for obstructive salivary lithiasis.

Our results can assist clinicians with the appropriate patient selection for sialendoscopy treatment. Additionally, they introduce a new question: When is the best time to indicate sialendoscopy in cases of obstruction due to strictures, where the main cause is inflammation (radioinduced, autoimmune sialodenitis)? Should it only be when they are symptomatic? Or should it be indicated early in the context of the disease? Perhaps more multi-center, prospective studies, with a greater sample size could address this question.

The main goal of the study is to apply these results in our daily clinic, selecting the better temporary moment to perform the procedure and not simply proposing the sialendoscopy act. Our results will help to choose the moment at which sialendoscopy will be indicated as the definitive treatment for obstructions by stones, preserving the gland and getting better QOL, or indicating as "palliative" treatment in cases of inflammatory strictures, expecting a poor improvement on QOL.

## Conclusions

Our study on post-sialendoscopy QOL found high score correlated with good patient satisfaction and overall good patient satisfaction after sialendoscopy in sialolithiasis, where 80.6% of symptoms improved.

We found a negative correlation between Xerostomia and post-sialendoscopy satisfaction, meaning poor QOL satisfaction perceived by the patient.

Our findings support the formal indication of sialendoscopy for obstructive sialolithiasis with a positive impact on QOL and probably a relative time-dependent indication for stenosis/other xerostomia causes that little improved QOL satisfaction.

## Data Availability

Not applicable.

## Abbreviations

QOL: Quality of life questionnaire
XER: Xerostomia degree questionnaire
OHIP: Oral health impact profile questionnaire
QoL: Quality of life questionnaires
CAAE: Institutional Ethics Committee
WHO: World Health Organization
UTN: Universal trial number
ReBec: Brazilian Clinical Trials Registry
PROCESS: Preferred reporting of case series in surgery
STROCSS: Strengthening the reporting of cohort studies in surgery
SQUIRE 2.0: Standards for Quality Improvement Reporting Excellence guidelines
VAS: Visual pain analog scale
Pre-VAS: Pre-sialendoscopy visual pain analog scale score
Post-VAS: Post-sialendoscopy visual pain analog scale score
PSPS: Questionnaire on patient satisfaction post-sialendoscopy
USG: Ultrasonography
